# Ketanserin as potential additive drug to improve V/Q mismatch in COVID-19?

**DOI:** 10.1186/s13054-020-03257-y

**Published:** 2020-08-28

**Authors:** M. Kuindersma, P. E. Spronk

**Affiliations:** 1 Department of Intensive Care Medicine, Gelre Hospitals, Albert Schweitzerlaan 31, Apeldoorn, The Netherlands; 2 Expertise center for Intensive care Rehabilitation Apeldoorn (ExpIRA), Apeldoorn, The Netherlands

**Keywords:** COVID-19, Coronavirus, Lung, Vasoconstriction, V/Q mismatch

To the editor,

With great interest we read the article by Santamarina et al. [1]. They describe possible underlying causes for the marked ventilation/perfusion (V/Q) mismatch in patients with COVID-19 while maintaining relatively normal lung compliance. In addition to platelet-fibrin thrombi in the small arterial vessels, they reported evidence of vasoconstriction on CT scans in the relatively unaffected lung parts causing profound V/Q mismatching. They postulate that this vasoconstriction is the effect of increased pulmonary angiotensin II activity with reduced cleavage by ACE-2 due to the SARS-COV2 infection.

In addition to microthrombi and increased angiotensin II activity, the V/Q mismatch may also result from microcirculatory alterations caused by local serotonin-induced arteriolar vasoconstriction. In non-hypoxic circumstances, serotonin can promote NO release from the endothelium via the 5HT_2b_ receptor and cause vasodilatation. In contrast, direct activation of smooth muscle cells (5HT_2a_ receptor) may cause vasoconstriction as well in a hypoxic environment. This milieu will induce increased NO consumption by erythrocytes. Consequently, stimulation via the 5HT_2b_ receptor in the endothelium will not yield further vasodilation due to low NO bioavailability, but will promote vasoconstriction of the smooth muscle tissue [2, 3]. In addition, serotonin secretion might be further increased by platelet activation in COVID-19, thus further enhancing microcirculatory vasoconstriction. This sequence of events may worsen the V/Q mismatch and hypoxia in COVID-19.

To improve oxygenation, patients are frequently ventilated in the prone position. The probable effect on oxygenation is an eversion of the vascular redistribution, and not alveolar recruitment. In addition to the relatively simple prone position, pulmonary vasodilators as NO and prostacyclin have been suggested in severe hypoxemia to improve V/Q mismatch in COVID-19 [4]. Unfortunately, until now, the role of ketanserin has been overlooked. This drug may reverse pulmonary microvascular vasoconstriction without major macrocirculatory effects, provided that adequate resuscitation has been applied [2]. This 5HT_2a_ receptor antagonist can inhibit vasoconstriction by serotonin in COVID-19, which benefits the perfusion of the relatively spared lung parts. In addition, the 5HT_2a_-mediated platelet inhibits aggregation, which inhibits thrombi formation and prevents further serotonin formation [5]. Given the combined effect of ketanserin on pulmonary vasoconstriction and platelet aggregation, it appears to be a valuable additive in the treatment of covid-19 and its use should be further explored.

## Data Availability

Not applicable.
